# The Path from Depressive Symptoms to Subjective Well-Being Among Korean Young Adults During the COVID-19 Pandemic: Mediating Roles of Housing Satisfaction, Social Capital, Future Achievement Readiness, and Occupational Hazards

**DOI:** 10.3390/healthcare13243189

**Published:** 2025-12-05

**Authors:** Miyoung Kwon, Myongsun Cho

**Affiliations:** 1 Department of Nursing, College of Health Science, Kangwon National University, Samcheok-si 25949, Republic of Korea; mykwon@kangwon.ac.kr; 2 Department of Nursing, Gangneung-Wonju National University, Wonju-si 26403, Republic of Korea

**Keywords:** Korean adult health, depressive symptoms, subjective well-being, housing satisfaction, social capital, future achievement readiness, occupational hazards

## Abstract

**Background:** Recent economic instability and social isolation have increased mental health vulnerabilities among young adults, highlighting the need to clarify how multiple contextual factors shape their subjective well-being. This study explored the relationship between depressive symptoms and subjective well-being among Korean young adults. It also investigated the mediating effects of housing satisfaction, social capital, occupational hazards, and future achievement readiness on this relationship. **Methods:** A parallel mediation model was used to analyze the mediating effects of housing satisfaction, social capital, future achievement readiness, and occupational hazards on the relationship between depressive symptoms and subjective well-being. The model examined direct and indirect pathways to determine the extent to which these factors influence subjective well-being in young adults. **Results:** Depressive symptoms were associated with reduced housing satisfaction, social capital, and future achievement readiness, as well as increased exposure to occupational hazards. All four variable associations between depressive symptoms and subjective well-being, suggesting that multiple structural and psychosocial conditions jointly shape young adults’ subjective well-being. **Conclusions:** The findings suggest that conventional mental health services alone may be insufficient. A multifaceted approach—including housing welfare policies, social connection support, employment and adjustment programs, and initiatives that enhance future preparedness—may help mitigate the negative effects of depressive symptoms and improve subjective well-being among young adults.

## 1. Introduction

### 1.1. Global and National Context

Young adulthood is a transitional developmental stage characterized by the establishment of self-identity and the acquisition of socioeconomic independence. However, these transitions also increase one’s vulnerability to mental health issues [1,2]. Recent social crises, such as the COVID-19 pandemic, have exacerbated housing insecurity, employment instability, and financial difficulties, which have further worsened the well-being and mental health of young adults [3].

Subjective well-being is multidimensional, encompassing emotional, psychological, and social aspects, and manifests differently in each individual [4]. It reflects an individual’s subjective assessment of life satisfaction, positive emotions, and life meaning, and can vary depending on personal values and situational context [4]. Subjective well-being plays a key role in young adulthood, enabling young adults to adapt to new environments and overcome challenges during life transitions (such as employment) in a healthy manner. By allowing young adults to pursue long-term goals and respond flexibly to failure, subjective well-being improves adaptability, fosters social relationships, and promotes self-growth, which enhances quality of life and mental health in adulthood [5]. Therefore, improving the subjective well-being of young adults is essential for their social integration and transition into healthy adulthood [6].

Existing studies on subjective well-being have focused on either middle-aged or older adults, addressing issues like population aging. Research on the subjective well-being of young adults remains relatively scarce [7]. This makes it imperative to identify the factors that influence their subjective well-being as well as the underlying mechanisms.

The age range for young adults varies by country. Some European countries have raised the upper age limit to 35 years in response to economic instability and changes in the pattern of entering adulthood [8]. In South Korea, the Framework Act on Youth serves as the legal foundation for youth-related policies. It defines young adults as individuals aged between 19 and 34 years [9]. This age range reflects the domestic situation in South Korea, where economic and social independence is delayed owing to factors such as longer education, employment instability, low incomes, and high housing costs [10].

High levels of depression are a major cause of reduced subjective well-being among young adults [11]. Negative cognitive biases reinforced by depression manifest as a tendency to evaluate oneself, others, and the future negatively, which hampers interpersonal relationships and self-concept [12]. When occurring repeatedly, such negative evaluations and emotional experiences reduce subjective well-being [12]. In South Korea, the number of individuals with depression increased from 752,000 in 2018 to 1 million in 2022. Annually, this number has increased by an average of 7.8%. In recent years, the rise has been even more pronounced, with a 22.8% increase among individuals in their 20s and a 13.7% increase among those in their 30s. Notably, these figures are higher than those observed in the overall [13].

The mental health of young adults should not be explained solely based on individual factors; it must be understood within a multidimensional context in which social and environmental factors interact [14]. Therefore, this study examined the association between depressive symptoms and subjective well-being among young adults and sought to determine whether housing satisfaction, social capital, future achievement readiness, and occupational hazards mediate this association individually or sequentially.

### 1.2. Literature Review

The pandemic-induced restrictions on young adults’ daily routines and social activities are reported to have increased depression and decreased life satisfaction [3,15]. Depression is not only known to diminish emotional well-being, but also to weaken future expectations and goal orientation [11,16]. These characteristics suggest that depression should be treated as a key antecedent to changes in young adults’ subjective well-being [17].

Young adults’ residential environments are known to be closely linked to life stability and subjective well-being [18,19]. Evidence shows that during the COVID-19 pandemic, factors like residential overcrowding, noise, and lack of privacy were associated with mental health [20]. Further, depression is known to be linked to cognitive biases that lead to a more negative perception of the external environment than reality [21]. Therefore, even under identical housing conditions, young adults with higher depression levels are likely to evaluate their housing satisfaction more negatively. Accordingly, this study hypothesized that depression potentially influences housing satisfaction.

Social capital, comprising social support, trust, and networks, is a key protective factor for youth well-being [19,22]. Consistent with this, reduced social contact during the COVID-19 pandemic was found to be associated with increased depression and decreased life satisfaction [14]. Furthermore, reduced social participation and weakened relationships were found to be correlated with higher depression levels [23], suggesting that depression may be linked to diminished social capital.

Future achievement readiness encompasses diverse resources such as educational attainment, parental socioeconomic background, personal effort, social relationships, and policy support [10]. Research has reported that these resource-based factors are associated with young people’s life satisfaction or well-being [19,24]. However, depression hinders future expectations and planning, weakening an individual’s ability to evaluate their resources positively [12], and can thus also affect their future-related judgments or perceptions of preparedness [11].

Job demands, employment instability, and low control among young adults are closely linked to life satisfaction [24,25]. Psychological symptoms like depression have been shown to affect perceptions of safety climate and risk judgment [26], with depressed individuals tending to interpret risk stimuli as more threatening than they actually are [22]. Furthermore, structural changes in the Korean youth labor market are acting as a backdrop increasing instability [16].

Depression is known to negatively impact young people’s cognitive, emotional, and social functioning overall. These effects can also be reflected in their evaluations of social and environmental resources, such as housing satisfaction, social capital, future preparedness, and perceptions of the work environment. These factors can change depending on an individual’s living conditions and policy environment, and are directly linked to youth well-being. Therefore, it is theoretically and empirically crucial to verify pathways starting from depression, through mediating factors with high intervention potential, to well-being, as this would contribute to enhancing youth well-being.

Furthermore, depression can influence not only these social and environmental factors but also cognitive processes such as how young people interpret and evaluate their future and resources [12,22,27,28], necessitating a more integrated understanding.

**Figure 1:**
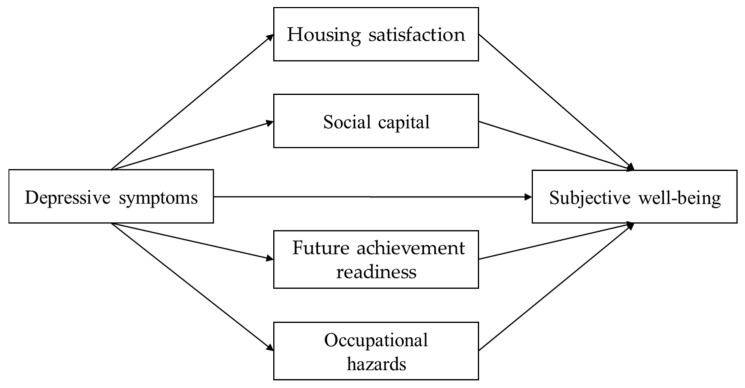
Hypothesized relationships among depressive symptoms, housing satisfaction, social capital, future achievement readiness, occupational hazards, and subjective well-being.

According to hope theory, depressive symptoms undermine motivation, goal orientation, and expectations of achievement, thereby reducing future achievement and consequently diminishing subjective well-being [17]. This perspective demonstrates that young adults’ well-being is shaped within a multidimensional context in which psychological, social, and environmental factors interact, highlighting the need for an integrated analysis. This approach suggests that understanding the impact of depressive symptoms, as well as the mediation of housing satisfaction, social capital, future achievement readiness, and occupational hazards, is necessary to comprehend young adults’ subjective well-being. Therefore, it is necessary to explore the pathways through which these socially and environmentally modifiable factors act in the relationship between depression and well-being. Accordingly, the following hypotheses were established (Figure 1).

**H1.**  *Depressive symptoms are associated with subjective well-being.*

**H2.**  *Housing satisfaction mediates the relationship between depressive symptoms and subjective well-being.*

**H3.**  *Social capital mediates the relationship between depressive symptoms and subjective well-being.*

**H4.**  *Future achievement readiness mediates the relationship between depressive symptoms and subjective well-being.*

**H5.**  *Occupational hazards mediate the relationship between depressive symptoms and subjective well-being.*

**H6.**  *Depressive symptoms influence subjective well-being through a sequential chain of four mediators—housing satisfaction, social capital, future achievement readiness, and occupational hazards.*

By testing these hypotheses, we aimed to identify the multidimensional factors influencing young adults’ subjective well-being and contribute to policy and practical interventions.

## 2. Materials and Methods

### 2.1. Data Source

We used raw data from the 2022 Survey on the Living Conditions and Welfare Needs of Youth Korea, which is conducted by the Korea Institute for Health and Social Affairs (KIHASA) [29] and published every two years according to the Framework Act on Youth. Considering the practical aspects of this survey, its sample excludes general households, island regions, dormitories, special social facilities, and foreign households. In 2022, the first year it was conducted, the survey included individuals aged 19 to 34 years (as of 1 January 2022) based on the 2020 Population Census (registered census), along with their household members. Using data from the 2020 Youth Statistics Register, 17 regions were stratified in two stages, and 250 cities, counties, and districts were selected using stratified probability-proportional-to-size sampling. Consequently, the 2022 National Survey on Youth Life targeted 15,000 general households.

### 2.2. Measurements

All variables in this study were measured using items derived from the standardized questionnaire developed by the KIHASA. This questionnaire was constructed through item development, expert review, and pilot testing processes to ensure reliability and validity [2].

#### 2.2.1. Dependent Variable

Subjective well-being was measured using three items concerning life satisfaction, current level of happiness, and freedom of choice in life. These items were rated on an 11-point scale [30]. The Cronbach’s alpha was 0.859.

#### 2.2.2. Independent Variable

Depressive symptoms were measured using the Patient Health Questionnaire-9 (PHQ-9), a self-report questionnaire developed by Spitzer et al. [31] as part of the Primary Care Evaluation of Mental Disorders. The PHQ-9 comprises nine items, each rated on a 4-point Likert scale ranging from 0 (“not at all”) to 3 (“every day”). The total score ranges from 0 to 27 points. According to Park et al. [32], 0–4 points denote no depressive symptoms, 5–9 points denote mild symptoms, 10–19 points denote moderate symptoms, and 20–27 points denote severe symptoms. The PHQ-9 has shown high reliability and validity among Korean adolescents [32]. The Cronbach’s alpha was 0.876.

#### 2.2.3. Mediating Variables

Housing satisfaction was measured using six items related to the place of residence at the time. These six items evaluated safety, rest, space for spending time with family, private space, physiological and hygienic functions (such as dining), and asset appreciation function. Each item was rated on a 5-point Likert scale ranging from 1 (“strongly disagree”) to 5 (“strongly agree”). The total score ranged from 6 to 30 points, and higher scores indicated greater satisfaction with the place of residence. The Cronbach’s alpha was 0.82.

Social capital consisted of three dimensions—social networks, community participation, and social trust—each measured using corresponding items [18,33]. Social networks were measured by quantifying the number of groups that could provide assistance in difficult situations (e.g., financial difficulties, living expenses, health issues, and stress), using five items rated on a 5-point scale. The Cronbach’s alpha was 0.849 for the sample in this study. The extent of community participation was measured using six items related to political and social participation (such as posting opinions online, signing petitions, and participating in protests) and three items regarding participation in community activities (such as regular participation in cultural, artistic, sports, or leisure activities over the past year). The Cronbach’s alpha for the nine items was 0.707. Social trust was measured using a single item rated on an 11-point scale: “To what extent do you feel that our society is trustworthy?” Furthermore, we verified that the three dimensions form the factor structure of social capital. To construct the social capital index, each dimension was first normalized using the formula (X − Xmin)/(Xmax − Xmin), rescaling the values to a consistent range between 0 and 1. To account for the varying influence of each dimension, we applied weights based on effect sizes and calculated a weighted average. This process yielded an index-type variable representing overall social capital [34].

Future achievement readiness was measured using six items concerning parents’ economic status, parents’ education level, one’s own education level, one’s effort, human networks, and government support policies. Respondents were asked to rate how well they were equipped to achieve their desired future on a 4-point Likert scale ranging from 1 (“Not at all equipped”) to 4 (“Fully equipped”). This variable was not merely a sum of objective resources; it encompassed subjective expectations and perceptions of policy-driven opportunity structures. Higher scores indicated that individuals possessed greater human and economic capital required to achieve their goals. This allowed the evaluation of young adults’ overall readiness for future achievements. The Cronbach’s alpha for the six items was 0.737.

Occupational hazards were measured using six items rated on a 5-point Likert scale ranging from 1 (“not at all”) to 5 (“every day”). The six items evaluated excessive workload, the hardship of working alone, carrying excessively heavy loads, pressure to achieve results, suppressing emotions during customer interactions, and insufficient safety and protective equipment. Higher scores indicated more occupational hazards. The Cronbach’s alpha was 0.793.

#### 2.2.4. Control Variables

Six covariates were included in the analytical model to test the robustness of the results. Specifically, the model’s effects were controlled for participants’ gender, age, employment status, education level, income, and perceived health. Gender was classified as male or female. Age was categorized into 19–24, 25–29, and 30–34 based on Korea’s policy-defined youth age classification [9] and developmental research, which suggests the transition to adulthood extends into the early 30s [35]. This distinction reflects the characteristics of Korean youth: prolonged educational pathways, delayed entry into the labor market, and delayed socioeconomic independence [2,25]. Employment status was categorized as either “employed” or “unemployed.” Education level was categorized as “high school or below,” “attending or on leave from university/college,” or “graduate or above.” Income was measured as the participant’s annual pre-tax income, including both wage income and business income, in thousands of KRW. Perceived health was measured on a 5-point Likert scale ranging from 1 (“very poor”) to 5 (“excellent”).

### 2.3. Data Analysis

We examined the factors affecting subjective well-being using two regression models. Model 1 included sex, age, employment status, education level, and income. Model 2 additionally incorporated perceived health, housing satisfaction, social capital, future achievement readiness, and occupational hazards. We verified the assumptions of regression analysis before testing, checked multicollinearity using variance inflation factors (VIF < 10), and evaluated the overall model fit using R^2^ values. Statistical significance was set at *p*-value < 0.05, and adjusted *p*-values were calculated using SPSS Statistics (version 27.0; IBM Corp, Armonk, NY, USA). We used PROCESS macro v4.2 (Model 4) to examine parallel multiple mediation, as well as assessed regression assumptions (normality, homoscedasticity, and independence) and evaluated multicollinearity using variance inflation factors (VIF). Indirect effects were tested using 5000 bootstrap samples to ensure robust inference.

## 3. Results

### 3.1. Sample Characteristics

The sample included 14,966 young adults, 52.1% of whom were female. The age distribution showed that the largest proportion of the sample (48.08%) was aged 19–24 years. Regarding employment status and education, most were employed (64.47%) and had a graduate degree or higher educational achievements (54.44%), respectively (Table 1).

### 3.2. Factors Affecting Subjective Well-Being

Table 2 presents the results of the linear regression analysis. In Model 1, sex (β = 0.05, *p* < 0.001), age (β = −0.05, *p* < 0.001), employment status (β = 0.03, *p* < 0.001), education level (β = 0.05, *p* < 0.001), and income (β = 0.08, *p* < 0.001) significantly predicted subjective well-being. In Model 2, perceived health (β = 0.15, *p* < 0.001), depressive symptoms (β = −0.27, *p* < 0.001), future achievement readiness (β = 0.21, *p* < 0.001), social capital (β = 0.14, *p* < 0.001), housing satisfaction (β = 0.12, *p* < 0.001), and occupational hazards (β = −0.09, *p* < 0.001) significantly predicted subjective well-being. The variance inflation factor ranged from 1.01 to 2.24, indicating the absence of multicollinearity. The Durbin-Watson statistic was 1.91 for Model 1 and 1.94 for Model 2, showing no autocorrelation among the independent variables. Model 1 explained 12% of the variance in subjective well-being, which increased to 31% in Model 2 (Table 2).

### 3.3. Mediation Analysis

**Figure 2:**
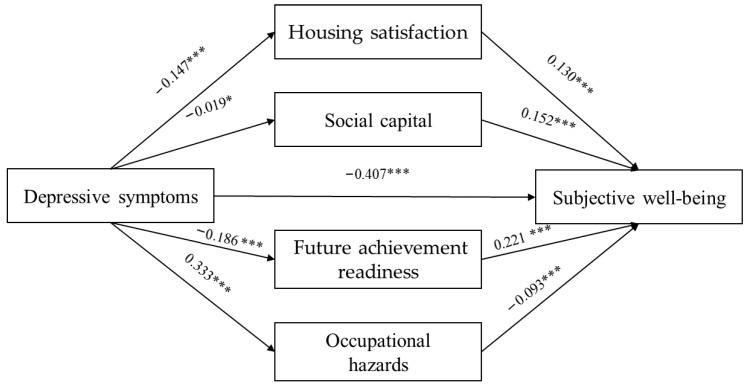
Hypothesized parallel mediation of housing satisfaction, social capital, future achievement readiness, and occupational hazards in the relationship between depressive symptoms and subjective well-being. ***Note*.** Effects were reported as standardized values. * *p* < 0.05, *** *p* < 0.001. X: Depressive symptoms, M1: Housing satisfaction, M2: Social capital, M3: Future achievement readiness, M4: Occupational hazards, Y: Subjective well-being.

Figure 2 illustrates the results of the parallel multiple mediation analysis testing the indirect effects of housing satisfaction, social capital, future achievement readiness, and occupational hazards in the relationship between depressive symptoms and subjective well-being. The results revealed the pathways through which depressive symptoms impact subjective well-being, mediated by housing satisfaction, social capital, future achievement readiness, and occupational hazards.

The detailed regression results for each mediation path are presented in Table 3. Depressive symptoms significantly predicted all four mediators—lower housing satisfaction (β = −0.150, *p* < 0.001), lower social capital (β = 0.020, *p* < 0.05), lower future achievement readiness (β = −0.190, *p* < 0.001), and higher occupational hazards (β = 0.333, *p* < 0.001). These mediators, in turn, were significantly associated with subjective well-being in the expected directions, supporting the hypothesized sequential relationships.

Table 4 presents the direct and indirect effects of depressive symptoms on subjective well-being, analyzed using 5000 bootstraps. The total effect was −0.184 (SE = 0.004, CI = −0.192 to −0.176), and the direct effect was −0.144 (SE = 0.004, CI = −0.152 to −0.136). The total indirect effect was −0.040 (SE = 0.003, CI = −0.045 to −0.035). Furthermore, the indirect effect of depressive symptoms on subjective well-being through housing satisfaction showed a coefficient of −0.009 (SE = 0.001, CI = −0.010 to −0.007). The indirect effect through social capital showed a coefficient of 0.001 (SE = 0.001, CI = 0.000 to 0.003). The indirect effect via future achievement readiness showed a coefficient of −0.019 (SE = 0.001, CI = −0.022 to −0.016). Finally, the indirect effect via occupational hazards exhibited a coefficient of −0.014 (SE = 0.002, CI = −0.017 to −0.011). These results indicated the partial mediation of housing satisfaction, social capital, future achievement readiness, and occupational hazards on the relationship between depressive symptoms and subjective well-being.

## 4. Discussion

This study empirically verified the impact of depressive symptoms on the subjective well-being of young adults and analyzed whether housing satisfaction, social capital, future achievement readiness, and occupational hazards mediate this relationship. The results revealed that the influence of depressive symptoms on young adults’ subjective well-being can be explained by four mediating variables. The proposed model explained 31% of the variance in subjective well-being, providing meaningful multifactorial explanatory power for understanding the subjective well-being of young adults. These findings suggest that policy support targeting modifiable factors—such as improving residential satisfaction, strengthening social capital, and alleviating occupational stress—can effectively enhance young people’s well-being. Therefore, the model developed in this study not only contributes theoretically, but practically as well by highlighting the importance of prioritizing practical strategies to promote youth mental health.

In this study, the overall level of depressive symptoms was low, with most participants falling within the non-depressed or mildly depressed range, consistent with previous studies reporting similar patterns among young adults [36]. These findings highlight the relative influence of structural (housing, work environment) and psychosocial (social capital, future readiness) factors in shaping subjective well-being. This evaluative perspective clarifies which domains may yield the greatest impact for public health strategies. Individuals with depression tend to evaluate themselves negatively and pay more attention to negative stimuli when processing environmental information, leading to negative responses to stimuli. This cognitive bias contributes to depression and reduces subjective well-being [22]. Given that the COVID-19 pandemic heightened uncertainty, social isolation, and emotional distress among young adults, the persistence of a direct pathway between depressive symptoms and subjective well-being may reflect enduring cognitive and emotional vulnerabilities during this period.

Depression was found to influence subjective well-being through housing satisfaction. Even in environments with identical physical conditions, including those of housing, individuals with higher depression levels tend to perceive their environment as more adverse [37]. With depression, individuals actively recognize inconveniences in their physical environment, including housing conditions [21]. During the COVID-19 pandemic, movement restrictions and prolonged indoor stay likely increased young adults’ exposure to their home environments, heightening the salience of housing conditions and potentially intensifying housing-related stress. This contextual factor may help explain the strengthened mediating role of housing satisfaction observed in this study. Similarly, in this study, housing-related stress appeared to exacerbate psychological vulnerability, thereby reducing subjective well-being. Research suggests that rent support and public rental housing improve housing satisfaction and quality of life by enhancing housing stability and reducing economic burden [38,39]. Therefore, economic support policies aimed at promoting housing stability and public housing supply may mitigate the effects of depression on young adults’ subjective well-being.

Social capital, such as social networks and community participation, was found to buffer the negative effects of depression on subjective well-being. Depression increases the tendency to avoid social situations or human relationships, which narrows one’s social networks [19]. Community participation satisfies basic psychological needs, such as autonomy, competence, and belonging, thereby increasing happiness [40]. Additionally, well-established social networks—networks that provide support in overcoming practical difficulties—have a significant effect on well-being [41]. During the COVID-19 pandemic, restrictions on in-person activities significantly limited opportunities for social interaction, which may have weakened many young adults’ social support systems. However, those with stronger pre-existing social capital may have maintained supportive relationships through digital or alternative communication channels, helping to buffer depressive symptoms during this period. Promoting community participation among young adults requires designs that reflect their needs. Therefore, it is essential to have a process where young people and researchers jointly reflect and create meaning of young adults’ diverse experiences [42]. Additionally, group living has been found to promote social support relationships, provide stress relief, alleviate social isolation, and enhance psychological stability [23]. Accordingly, building youth-friendly social infrastructures—such as community centers, hybrid offline–online participation platforms, and relationship-building programs—may strengthen social networks and help mitigate the mental health burden of depression. These findings reinforce the importance of community-driven public health approaches that build supportive environments and reduce social exclusion among youth.

Future achievement readiness was found to mediate the relationship between depression and subjective well-being. Parents’ socioeconomic backgrounds, one’s economic and educational resources, social networks, and government support policies all interact and shape young adults’ future achievement readiness, which showed a direct influence on subjective well-being and depression risk. This finding is consistent with those of previous studies highlighting socioeconomic inequality as a core social factor directly linked to young adults’ mental health [25,43]. Depression and well-being are more severe in groups with low socioeconomic resources, suggesting that social structural discrimination continues and causes them to have a negative outlook on their life choices [44]. During the COVID-19 pandemic, disruptions in academic progression, reduced employment opportunities, and prolonged uncertainty may have heightened concerns about future prospects for many young adults, potentially reinforcing the mediating role of future achievement readiness observed in this study. This suggests that, in addition to providing mental health support, comprehensive social safety nets and targeted socioeconomic support measures are urgently needed to improve future outlooks among socioeconomically disadvantaged young adults. This underscores the need for higher education institutions and public health systems to expand career guidance, academic support, and socioeconomic assistance programs that can reduce future uncertainty among vulnerable groups.

Occupational hazards are more common among individuals with depression, and they were found to hamper subjective well-being. Individuals with depression are more likely to be exposed to hazardous work environments because of symptoms such as fatigue, reduced reaction speeds, and impaired judgment [26]. They are also more likely to face unemployment, long-term absence, and work disabilities, which negatively impact employment opportunities and increase exposure to dangerous and poor working conditions [45]. During the COVID-19 pandemic, young adults—particularly those employed in temporary, part-time, or high-risk occupations—experienced heightened job insecurity and increased exposure to unsafe working environments, which may have further intensified the link between depression, occupational hazards, and reduced well-being. An organizational approach is essential to improve the hazardous working conditions of young adults with depression. Enhancing the quality of employer–employee relationships and proactive work adjustments, such as flexible work arrangements and job modifications, are beneficial for individuals with depression [24]. Despite their abundance, individuals in transitional life stages have limited access to mental health services [46]. To address this issue, implementing easily accessible digital platforms and co-developing mental health services with young people may be effective [46]. Expanding low-barrier digital mental health platforms and co-developing youth-centered services may help address these gaps. However, to deliver such organizational and government-level services, it is necessary to first establish policies for their implementation and maintenance.

Taken together, these findings highlight the importance of addressing multiple structural and psychosocial factors simultaneously to improve the well-being of young adults. Rather than focusing on a single domain, youth mental health policies should integrate housing stability, opportunities for social engagement, educational and employment support, and accessible mental healthcare into a coordinated system. The COVID-19 pandemic revealed how fragile these systems can be, underscoring the need for sustainable infrastructures that protect young adults during periods of social and economic disruption. Moreover, the pathways identified in this study offer practical implications for both public health and higher education. Universities can incorporate mental health support, digital readiness training, and inclusive campus environments, while policymakers and community leaders can strengthen digital infrastructure, reduce social inequities, and expand youth-centered support systems. These efforts may also help reduce digital and AI competency gaps that increasingly shape young adults’ academic and economic opportunities.

This study has several limitations. First, because the data were collected at a single point in time, causal relationships among the variables could not be established definitively. Second, although the validated multi-item scales developed by KIHASA were used, reliance on self-reported data introduces the possibility of subjective or recall-related bias. Third, the study did not examine additional mediating or moderating factors—such as resilience, social comparison, or digital media use—that could provide a more comprehensive understanding of the pathways linking depressive symptoms and subjective well-being. Future research should employ longitudinal designs and incorporate data from multiple sources to strengthen temporal and cultural validity. Furthermore, because the study was conducted during the COVID-19 period, seasonal factors and pandemic-related stressors may have influenced mental health indicators. Therefore, long-term follow-up studies that better account for contextual influences are necessary.

## 5. Conclusions

This study comprehensively verified the direct and indirect pathways through which depressive symptoms affect subjective well-being among young adults. By doing so, it found that youth-centered policies—such as those promoting housing stability, social capital, and future achievement readiness—can help effectively protect young adults’ mental health and enhance their subjective well-being. Depressive symptoms were identified as a major factor lowering subjective well-being, and housing satisfaction and future achievement readiness were found to be key mediators in this relationship. In contrast, social capital was found to have a buffering moderating role. Therefore, it is necessary to develop policies that integrate mental health services with social policies such as housing welfare, employment support, and adaptation assistance in depression interventions. Future studies should use longitudinal designs and multi-source data to verify causal relationships, evaluate intervention effects, and refine youth mental health interventions.

## Figures and Tables

**Table 1:** Characteristics of the participants (*N* = 14,966).

Variable	Categories	N (%) or M ± SD
Sex	Male	7171 (47.90%)
	Female	7795 (52.10%)
Age	19–24 years	7195 (48.08%)
	25–29 years	4549 (30.40%)
	30–34 years	3222 (21.53%)
Employment status	Unemployed	5317 (35.53%)
	Employed	9649 (64.47%)
Education level	High school or below	2084 (13.92%)
	Attending or on leave from university/college	4734 (31.63%)
	Graduate or above	8148 (54.44%)
Depressive symptoms	Normal	11,848 (79.2%)
	Mild depressive symptoms	2253 (15.1%)
	Moderate depressive symptoms	748 (5.0%)
	Severe depressive symptoms	117 (0.8%)
Annual income (KRW 0–35,000)		1917.26 ± 1784.01
Perceived health (1–5)		3.6 ± 0.79
Housing satisfaction (0–48)		24.04 ± 4.61
Social capital (0–1)		0.38 ± 0.2
Future achievement readiness (6–24)		16.06 ± 2.36
Occupational hazards (6–30)		10.11 ± 4.71
Subjective well-being (0–10)		6.85 ± 1.74

Note: M = mean, SD = standard deviation.

**Table 2:** Results of identifying the factors associated with subjective well-being (N = 14,966).

	Model 1				Model 2			
Variable	B	SE (B)	β	t	B	SE (B)	β	t
Sex (male)	0.16	0.03	0.05	4.82 ***	0.19	0.03	0.05	6.32 ***
Age	−0.11	0.02	−0.05	−4.63 ***	−0.02	0.02	−0.01	−1.04
Employment status	0.19	0.06	0.03	3.19 ***	0.15	0.05	0.02	2.90 *
Education level	0.11	0.02	0.05	4.94 ***	−0.03	0.02	−0.01	−1.25
Income	0.00	0.00	0.08	7.50 ***	0.00	0.00	0.06	6.59 ***
Perceived health					0.34	0.02	0.15	16.97 ***
Depressive symptoms					−0.12	0.00	−0.27	−28.77 ***
Future achievement readiness					0.15	0.01	0.21	23.95 ***
Social capital					1.23	0.07	0.14	16.68 ***
Housing satisfaction					0.05	0.00	0.12	14.30 ***
Occupational hazards					−0.03	0.00	−0.09	−10.30 ***
Adjusted R^2^	0.12				0.31			
F	238.37 ***				106.04 ***			
Durbin-Watson	1.91				1.94			

Note. * *p* < 0.05, *** *p* < 0.001; SE = standard error.

**Table 3:** Results of mediation analysis.

		B	SE (B)	t	β	F(R^2^)
Housing satisfaction	Depressive symptoms	−0.175	0.010	−15.210	−0.150	231.29 *** (0.02)
Social capital	Depressive symptoms	0.001	0.001	1.974	0.020	3.9 * (0. 01)
Future achievement readiness	Depressive symptoms	−0.114	0.006	−19.608	−0.190	384.49 *** (0.04)
Occupational hazards	Depressive symptoms	0.404	0.011	36.323	0.333	1319.37 *** (0.11)
Subjective well-being	Depressive symptoms	−0.144	0.004	−35.798	−0.318	845.84 *** (0.29)
	Housing satisfaction	0.049	0.003	15.393	0.130	
	Social capital	1.347	0.075	18.032	0.152	
	Future achievement readiness	0.165	0.006	25.567	0.221	
	Occupational hazards	−0.035	0.003	−10.592	−0.093	
Subjective well-being	Depressive symptoms	−0.184	0.004	−45.700	−0.407	2088.46 *** (0.17)

*Note.* * *p* < 0.05, *** *p* < 0.001.

**Table 4:** Direct and indirect effects of depressive symptoms on subjective well-being, analyzed using 5000 bootstraps.

Path		Coeff	SE	LLCI	ULCI
Total effect		−0.184	0.004	−0.192	−0.176
Direct effect		−0.144	0.004	−0.152	−0.136
Indirect effect	Total	−0.040	0.003	−0.045	−0.035
	X→M1→Y	−0.009	0.001	−0.010	−0.007
	X→M2→Y	0.001	0.001	0.000	0.003
	X→M3→Y	−0.019	0.001	−0.022	−0.016
	X→M4→Y	−0.014	0.002	−0.017	−0.011

*Note*. X: Depressive symptoms, M1: Housing satisfaction, M2: Social capital, M3: Future achievement readiness, M4: Occupational hazards, Y: Subjective well-being, Coeff: coefficient, SE: Standard error, LLCI: Lower level of 95% confidence interval, ULCI: Upper level of 95% confidence interval.

## Data Availability

The data presented in this study are openly available in Korea Institute for Health and Social Affairs at https://mdis.kostat.go.kr/dwnlSvc/ofrSurvSearch.do?curMenuNo=UI_POR_P9240 (accessed on 18 April 2025) with permission.
